# Contribution to the Study of Congenital Syphilis

**Published:** 1870-02

**Authors:** W. H. Van Buren

**Affiliations:** Professor of Principles of Surgery, with Diseases of Genito-Urinary Organs, Bellevue Hospital Medical College


### CONTRIBUTION TO THE STUDY OF CONGENITAL SYPHILIS.

By W. H. VAN BUREN, M.D., Professor of Principles of Surgery, with Diseases of Genito-Urinary Organs, Bellevue Hospital Medical College.

The scientific interest, always inspired by congenital syphilis, and its unsolved problems, arises from the grave necessity for accurate knowledge of the subject, which is daily felt by the physician in dealing with the tenderest interests of humanity. The serious and complex question as to the propriety of marriage, the best mode of rearing infected offspring, the chances of their ultimate and entire restoration to health, the remoter consequences of congenital poisoning, are amongst the many difficulties which are yet awaiting the positive solution that science requires. This result is to be attained, not by polemical discussion and criticism of disputed doctrines, but rather by striving to see clearly and to note truthfully the facts which are daily passing before our eyes, content to sacrifice personal predilections, and to leave the result to the future. It is not given to every man to generalize, certainly not to generalize correctly, from insufficient data. But there are few practising physicians who cannot furnish the details of some facts which have fallen within the scope of their own observation and experience, bearing upon these unsolved problems; and it is from such facts as these that the science of the future is to be built up—slowly and laboriously, perhaps, but surely and certainly.

In this spirit, I contribute the following history, which, although meagre in some of its details, I have endeavored to relate as accurately as the circumstances have permitted:—

At the age of 20, B. had chancre in Philadelphia, in the winter of 1854–55, for which he took mercury for three weeks, and was slightly ptyalized. The next year he had chancres in Florence. In the succeeding year he contracted gonorrhœa in Paris, which was followed by buboes in both groins, with suppuration on one side, and by warts. In the spring of 1857, he had chancre again, for which he took mercury for a month. In the autumn of 1857, he called upon me for an opinion as to the propriety of matrimony. The Florentine chancres had destroyed about one-half of the glans penis; and it was this deformity alone that occasioned the doubt in reference to which I was consulted, as I was told on enquiring as to the existence of any symptoms of constitutional syphilis. He had no anxiety on this score, having never been conscious of any secondary symptoms, and refused entirely to entertain the idea of constitutional infection, of which, indeed, he presented no external evidence, being apparently in robust health. I decided that the deformity of the penis was no impediment to marriage, and he declined any further examination. After this he was not exposed to the risk of any new contagion. In August, 1858, he married a perfectly healthy party, never before married, and, at the end of nine months and seventeen days, his wife was delivered of a healthy female child, who is still living, and who has always enjoyed robust health.

In June, 1860, a second female child was born, with defective development of the brain and spinal cord. This child, as far as I can learn, never manifested any of the ordinary evidences of congenital syphilis. It showed a great deal of tenacity of life, although idiotic and paraplegic, and died at the age of five, from the consequences of whooping-cough.

In October, 1860, I was consulted in regard to the wife and the second child. The former, from the enjoyment of brilliant health, at the time of her marriage, had become gradually pale, weak, and emaciated, without obvious cause. She had nursed both of her children. I was unable to make out any symptoms of secondary syphilis, save a characteristic ulcer upon the side of the tongue, of at least six months' duration; she had also a gummy tumor, the size of a hickory nut, in the left LABIUM MAJUS. These symptoms disappeared, and her health rapidly improved under the use of the bi-chloride of mercury and the iodide of potassium, which were continued for more than a year, and pretty regularly, as far as I can learn, under the supervision of a highly intelligent physician, in a distant city.

In 1861, I was consulted by the husband for amblyopia, and referred him to my friend, Dr. Agnew, who recognized "ophthalmoscopic signs of RETINITIS SYPHILITICA, of a mild type, and distinct indelible retinal lesions, such as patches of proliferated tissue and atrophy." These were the first evidences of constitutional syphilis he had manifested. These syphilitic lesions were also complicated with those of AMBLYOPIA POTATORUM. He was advised to use the bi-chloride of mercury, which he did, somewhat irregularly, for several months, and, as I satisfied myself at a later period, with great benefit to his sight. This, indeed, he also recognised, for he resumed its use, subsequently, of his own accord, on several occasions.

In January, 1862, another child was born (a boy), who, as I learned from the family physician, in a letter dated April 9, 1862, "appeared quite healthy at birth, and continued so until three weeks ago, when an eruption made its appearance, complicated with excoriations around the mouth and nose, which gradually invaded the whole body, and was," in the writer's opinion, "decidedly syphilitic." He adds: "There are at present no marks of morbid action in the mother, but she has resumed the use of the corrosive sublimate, and is nursing the child." The boy was treated by the hydarg. c. creta, for a few weeks, and the symptoms detailed above disappeared, but he remained pale and delicate. In February, 1866, the beginning of his fifth year, he began to suffer from nocturnal pains in both tibiæ, and this symptom was shortly followed by periostal swelling, involving about three inches of the subcutaneous surface of both bones. He was brought to me in the autumn of this year, when I verified these symptoms, and ascertained, also, that the boy had been affected by complete paraplegia, which had continued but for one day, on two different occasions, and that he habitually suffered from irritability of the bladder, requiring to be taken up two or three times every night. He had also frequent attacks of pain through the nose, at the base of the cranium, and elsewhere. I found enormous nodes on both shins, and another on the subcutaneous surface of the right ulna. He was pallid, irritable, dejected, easily fatigued, dainty and capricious in his appetite, and sleeping badly from nocturnal pains. No specific treatment had been employed since the first three months of his life.

The mother was in apparently perfect health, with an excellent color and robust appearance, and had been in this condition for three years. The first born daughter, whom I also saw at this time, was a model of health. The boy was subjected, at once, to the systematic treatment of cod liver oil and the iodide of potassium, in doses of two grains, afterwards doubled. All of his symptoms gradually disappeared within the month, and he left the city immensly improved in appearance and strength, some prominence of the tibiæ alone remaining. After this the remedies were employed irregularly, and he became more delicate again. At the period of second dentition, I learn from his dentist, in a letter dated October, 1869, that he found "unmistakable evidences of the disease upon his incisor teeth, the development of the enamel quite imperfect—in fact, what I call syphilitic teeth."

About this date, the father again came under my observation, suffering from irregular, nodulated, and tender shins, superficial ulcers at the angles of the mouth, a yellowish patch on one tonsil, and a circular scaly spot, as large as a sixpence, on one of his palms. He had been exposed to no new infection. These symptoms commenced to yield promptly to mixed treatment. His general health is vigorous.

I learn that another child was born to him in October, 1867, the mother's health having continued excellent up to this period, when an ovarian enlargement was detected, which led to a fatal complication in the form of peritonitis, some weeks after delivery. This child survives in excellent health, having shown no evidences whatever of disease. The boy, now seven years of age, is still delicate, suffering occasionally from pains in his legs.

In this history, a sound, healthy woman is married to a man of unusually vigorous constitution, certainly affected with the syphilitic diathesis, but not aware of it, and they have four children. The first is healthy, the second idiotic, the third syphilitic, the fourth healthy; the mother having acquired the disease, and anti-syphilitic treatment having been employed, by which she was apparently cured, the father showing evidences of the disease. The mother acquired the disease during the first pregnancy and lactation, and as the child is healthy, this contamination was probably an example of direct secondary contagion, the alternative being that she was poisoned by a child who has never shown any evidences of disease. The second child was born, both parents now suffering from the disease, with arrested development of the cerebro-spinal axis, but showing none of the ordinary evidences of congenital syphilis. Was this arrest of development due to the syphilitic poison?

A third child was born after both parties had been subjected to anti-syphilitic treatment, but apparently not to an extent to save their offspring from infection. The disease in this child, assisted by treatment, passed through its secondary stage, to reappear four years later in its tertiary form; and here it assumed that somewhat rare phase of infantile syphilis in which the osseous system is involved.* It is noticeable that in this child there has been no sign of scrofula.

After further perseverence in anti-syphilitic treatment, the mother seems to have passed through the tertiary stage of the disease, and, apparently, to have recovered her health entirely. The father, less steady in the use of remedies, is not so happy, having manifested, still later, advanced secondary symptoms, unmistakeable in character. Under these circumstances, another child, in all respects healthy, is born to them.

It is worthy of remark that the father, with no obvious signs of syphilis, or even of cachexia, communicates to the wife disease, with marked cachexia, which rapidly runs into its final stage and apparently gets well; the diathesis more firmly established in him, yet milder and more chronic in its subsequent manifestations, still persisting. Also, that the mother, with tertiary symptoms, gave birth to a son who had secondary symptoms, the father having not yet passed the secondary stage; and that his son had, subsequently, tertiary manifestations. Finally, that the mother had no abortion or miscarriage, and nursed all her children.—AMERICAN JOURNAL OF SYPHILOGRAPHY.

### THE CLIMATE WHICH CURES CONSUMPTION.

A series of valuable articles have appeared in the MEDICAL TIMES AND GAZETTE, on the climate of the Peruvian Andes.

We will suppose our readers acquainted, to some extent at least,

* A case which I observed, in 1853, of congenital syphilis, involving the osseous system, has been recorded by my friend, Prof. Bumstead, in his excellent work on venereal diseases, p. 465.

